# Cost-Effectiveness of Cerebrolysin after Ischemic Stroke: Secondary Analysis of the CARS Study

**DOI:** 10.3390/healthcare11101497

**Published:** 2023-05-21

**Authors:** Stefan Strilciuc, Constantin Radu, Diana-Alecsandra Grad, Adina Dora Stan, Cristian Vladescu, Anca Dana Buzoianu, Dafin Muresanu

**Affiliations:** 1 Department of Neuroscience, Iuliu Hatieganu University of Medicine and Pharmacy, 400012 Cluj-Napoca, Romania; stefan.strilciuc@ssnn.ro (S.S.);; 2 RoNeuro Institute for Neurological Research and Diagnostic, 400364 Cluj-Napoca, Romania; 3 Department of Public Health, Faculty of Political, Administrative and Communication Sciences, Babes-Bolyai University, 400084 Cluj-Napoca, Romania; 4 Yale School of Public Health, Yale University, New Haven, CT 06520, USA; 5 National Institute for Management of Health Services, 021254 București, Romania; 6 Faculty of Medicine, Titu Maiorescu University, 031595 București, Romania; 7 Department of Clinical Pharmacology, Iuliu Hatieganu University of Medicine and Pharmacy, 400012 Cluj-Napoca, Romania

**Keywords:** stroke, cerebrolysin, cost-effectiveness

## Abstract

The cost-effectiveness of Cerebrolysin as an add-on therapy for moderate–severe acute ischemic stroke is a topic that remains understudied. This study aims to address this gap by performing a comprehensive cost-utility analysis using both deterministic and probabilistic methods from a payer perspective and within the Romanian inpatient care setting. Quality-adjusted life years (QALYs) were calculated using partial individual patient data from the 2016 Cerebrolysin and Recovery After Stroke (CARS) trial, utilizing three different health state valuation models. Cost data was extracted from actual acute care costs reported by Romanian public hospitals for reimbursement purposes for patients included in the CARS study. Incremental cost-effectiveness ratios were calculated for each treatment arm for the duration of the clinical trial. Deterministic analysis based on sample mean values indicates Cerebrolysin would be cost-effective at a threshold between roughly 18.8 and 29.9 thousand EUR, depending on valuation techniques. Probabilistic sensitivity analysis results indicate an 80% chance probability of cost-effectiveness of Cerebrolysin as an add-on therapy for acute ischemic stroke, considering a willingness-to-pay threshold of 50,000 EUR in a 90-day timeframe after stroke. Further economic evaluations of Cerebrolysin are needed to strengthen these findings, covering a timeframe of at least 12 months after the acute incident, which would account for treatment effects spanning beyond the first 90 days after ischemic stroke. These should be conducted to determine its cost-effectiveness under various care settings and patient pathways. Most importantly, modelling techniques are needed to answer important questions such as the estimates of population gain in QALYs after acute administration of Cerebrolysin and the potential offsetting of direct medical costs as a result of administering the intervention.

## 1. Introduction

Stroke continues to be one of the leading death and disability causes worldwide, with 12.2 million new cases (among which 62.4% were ischemic) and 6.55 million deaths in 2019 [1]. A major public health issue, the stroke is projected to hold its global rank by 2040 on the list of causes for years of life lost [2]. Acute management of stroke has progressed significantly in the past decades, resulting in a considerable reduction in mortality rates [3], while advances in neurorehabilitation have led to improvement in outcomes after stroke [4]. To alleviate the global burden of stroke, continuous expansion of care networks, improvements in care standards and proper implementation of primary and secondary prevention guidelines are required [5,6]. The changing age distribution of populations and increasing economic burden of innovation with health technologies have powerful implications for both the supply and demand of healthcare services [7,8]. The COVID-19 pandemic has caused major disturbances in this already fragile equilibrium by redirecting resources and redesigning health services delivery priority [9,10], generating a tsunami of unforeseen health care needs for both communicable and non-communicable diseases and creating consequential setbacks in most other disease areas [11]. An increasing body of evidence points out that stroke admissions have substantially decreased during the pandemic [12,13,14,15] and measured time of stroke care acute indicators (door-to-imaging, door-to-needle, door-to-groin, door-to-reperfusion) for assisted cases have increased [16].

The ability of countries to simultaneously navigate crises and tackle long-term challenges is largely dependent on socioeconomic context [17], as impoverished health systems are less equipped to focus on the shifting health needs of the population [18]. In the context of low health expenditure, circumstantial resource allocation and poor mechanisms for health technology assessment often considerably hinder improvement in populational health. As a result of this constellation of determinants, health systems are under unprecedented distress, while being forced to operate under budgetary constraints similar to the pre-pandemic period. The importance of developing and incorporating cost-effectiveness evidence in health decision making and resource allocation is becoming paramount, even as the world prepares to return to “normal” once COVID-19 reaches an endemic phase [17].

The landscape of stroke care is rapidly evolving, and the incorporation of novel therapeutic approaches and technologies is essential to improve patient outcomes and reduce the burden on healthcare systems. With an aging population and an increase in the prevalence of risk factors, such as hypertension, obesity and diabetes, the demand for efficient and cost-effective stroke treatments will continue to rise [1]. In addition, the complexity and interrelated nature of stroke pathophysiology and treatment necessitates a multidisciplinary approach, which can prove challenging in the context of constrained resources and competing healthcare priorities [19].

As healthcare systems worldwide strive to optimize the allocation of resources and improve patient outcomes, cost-effectiveness analysis plays a crucial role in informing policy decisions and clinical practice guidelines [20]. The integration of economic evaluations into the decision-making process can help identify the most efficient use of limited healthcare resources and facilitate the adoption of new therapies that provide the greatest value to patients and society [21]. Given the significant human and economic impact of a stroke, the assessment of cost-effectiveness for emerging therapies is of particular importance to ensure that healthcare systems can provide optimal care while maintaining financial sustainability [22].

Cerebrolysin is a promising biological agent that mimics the biological action of neurotrophic factors, aiding both neuroprotection and neurorecovery after ischemic stroke. It has been recently included in several clinical guidelines [23,24,25] and is particularly recommended as an add-on treatment in moderate–severe cases. Cerebrolysin is a peptide-based medication derived from porcine brain tissue, which contains a mixture of neurotrophic factors and active peptides that demonstrate neuroprotective and neurorestorative properties. The mechanism of action of Cerebrolysin is multifaceted, involving reduced inflammation, decreased apoptosis, enhanced neurogenesis and increased synaptic plasticity [26]. These combined effects contribute to the improvement of neurological outcomes and functional recovery in patients with ischemic stroke. Several clinical trials have demonstrated the efficacy and safety of Cerebrolysin as an adjunct therapy to standard stroke care, with better functional outcomes and lower rates of adverse events compared to placebo [27,28]. Cerebrolysin has shown potential benefits in other neurological disorders, such as Alzheimer’s disease and traumatic brain injury [29,30,31]. Although it is emerging as part of care standards, there is little evidence regarding the cost-effectiveness profile of Cerebrolysin [32,33].

Our research seeks to expand current knowledge on this topic by providing a new cost-utility assessment on Cerebrolysin as complementary treatment for moderate to severe ischemic stroke. Understanding the agent’s cost-effectiveness profile is critical to inform decision-makers and clinicians of the potential value it could provide in the management of ischemic stroke. As healthcare systems grapple with the challenges of delivering high-quality care under budgetary constraints, the generation of robust cost-effectiveness evidence for Cerebrolysin can help support its integration into clinical practice and optimize the use of limited resources.

Moreover, the examination of cost-effectiveness in the context of the Romanian healthcare system is particularly relevant, given its unique challenges and the need for evidence-based decision-making. Romania faces considerable disparities in access to healthcare services, particularly in rural areas, and is characterized by a high degree of fragmentation in service provision [34]. In addition, the country has one of the lowest healthcare expenditures in the European Union, resulting in a significant unmet need for quality care [35]. By conducting a cost-utility analysis of Cerebrolysin within the Romanian inpatient care setting, this study aims to provide valuable insights for policymakers and healthcare providers in addressing the specific needs and constraints of the local context. Furthermore, the secondary analysis of the CARS study offers a unique opportunity to assess the cost-effectiveness of Cerebrolysin using real-world data from a well-designed clinical trial. By leveraging individual patient data and actual acute care costs reported by Romanian public hospitals, the findings of this study can help bridge the gap between clinical efficacy and the economic implications of Cerebrolysin as an add-on therapy for moderate–severe acute ischemic stroke.

This study seeks to provide a comprehensive assessment of the cost-effectiveness of Cerebrolysin in the context of the Romanian healthcare system, contributing to the evidence base for its potential value in improving stroke outcomes and informing healthcare decision-making. By combining clinical, economic and policy perspectives, our research aims to shed light on the complex interplay between stroke care, resource allocation and health outcomes, ultimately providing valuable insights for the ongoing development and refinement of stroke management strategies.

## 2. Materials and Methods

**Figure 1:**
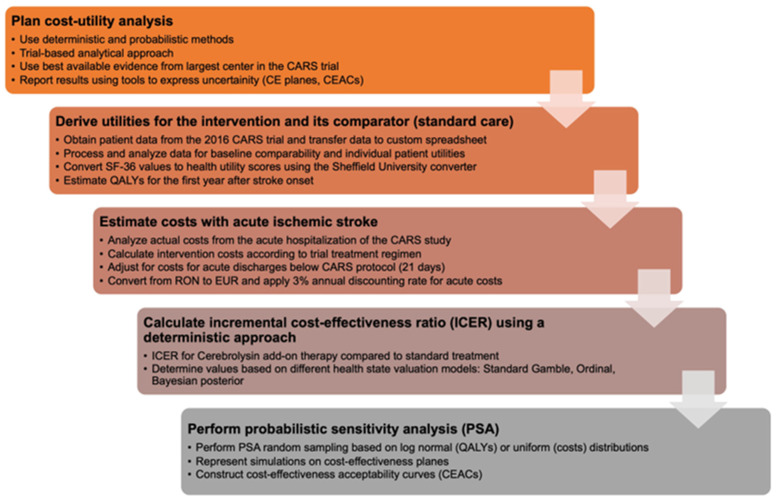
Methodological flow describing steps taken in the analysis. CE: Cerebrolysin, CEACs: Cost-effectiveness acceptability curves, CARS: Cerebrolysin and Recovery After Stroke, QALY: Quality-adjusted life years.

Using deterministic and probabilistic approaches, we conducted a cost-utility evaluation to compute the incremental cost-effectiveness ratio (ICER) for Cerebrolysin, drawing on information gathered from multiple sources. Deterministic methods (using fixed input values) in economic evaluations involve calculating cost-effectiveness using single-point estimates for all input parameters, such as costs, effectiveness and utilities. This approach does not account for uncertainty or variability in the input parameters, and thus, the results may not reflect the full range of possible outcomes. Probabilistic methods (incorporating uncertainty) in economic evaluations, on the other hand, use probability distributions for input parameters to account for uncertainty and variability. By performing multiple simulations, these methods generate a range of possible outcomes, providing a more comprehensive understanding of the cost-effectiveness and the likelihood of different scenarios occurring. Due to the nature of data collection (secondary analysis of clinical trial data; extraction of anonymized and aggregated cost values for various care scenarios), Institutional Review Board approval was not required for this study. To simulate patient pathways and costs, we selected the main care setting from the only available clinical trial that may be used to derive utilities for inpatient acute ischemic stroke care, based on the dominant participating country (Romania). This analysis was performed from the perspective of the payer, based on a predefined sequence (Figure 1).

### 2.1. Deriving Utilities for the Intervention and Standard of Care

We obtained individual patient data from the 2016 Cerebrolysin and Recovery After Stroke (CARS) trial from the principal investigators [36]. Patient demographics, medical history, risk factors and 36-Item-Short-Form-Survey (SF-36) results (*n* = 208) were transferred to a Microsoft Excel workbook and further processed and analyzed to assess baseline comparability and to obtain individual patient utilities for Cerebrolysin in the parallel quasi-experimental paradigm. SF-36 data consisted of single score values at day 2 and day 90 after stroke onset (visit 2 and visit 7, respectively). The resulting database included 198 patients. SF-36 values were converted to health utility scores using converters [37] developed by School of Health and Related Research (ScHARR) at the University of Sheffield. Several instances of missing SF-36 data were handled by excluding cases with missing values on more than 3 SF-36 subscales on either visit (*n* = 10) or by recoding missing entries as 9 (*n* = 13), according to the instructions of the SF-36 to SF-6D utility score converter. The tool generated three different types of utility scores using standard gamble, ordinal and Bayesian posterior mean health state valuation models. Due to missing values, the converter excluded an additional 5 cases, resulting in 193 patients included in the final analysis (100—Cerebrolysin; 93—placebo).

The utility scores generated were used to calculate quality-adjusted life years (QALYs) for the first year after stroke onset. Three values were obtained for each patient based on the health state valuation models used in the convertor tool. The formula used to calculate QALYs for each individual patient was:

$$\left(\left(2\;*\;D2+88\;*\;AVERAGE\;\left(D2,\;D90\right)\right)\;/\;90\right)\;*\;0.25$$

where *D*2 is the utility score for day 2 and *D*90 is the utility score for day 90. The assumptions within this QALY calculation fit the definitions and timeline for stroke recovery set by Bernhardt et al. (2017), where improvement is registered in the acute and early subacute phases (up to day 90), followed by a plateau in neurorecovery [38].

### 2.2. Extracting Costs with Acute Ischemic Stroke

Cost data on inpatient standard treatment of stroke was determined by analyzing actual costs from the acute hospitalization of the CARS study. Reported sums include direct (staff wages, medication, supplies, diagnostic services) and indirect (administration and overhead) hospital costs associated with inpatient care. Costs were converted from RON to EUR based on an average conversion rate between 15 November and 15 December 2021 of 1 EUR = 4.95 RON and rounded to the nearest integer value. The cost for one therapeutic unit (10 mL) of Cerebrolysin was retrieved from the webpage of the Romanian National Health Insurance House (5.5 EUR per 10 mL vial). This cost represents the maximal price set by national legislation and covers the most expensive scenario. Given that the treatment course used in the CARS study consisted of 30 mL of Cerebrolysin per day, diluted with physiological saline solution to a total volume of 100 mL, we added the cost of a 100 mL vial of saline solution (0.6 EUR) to the cost of three Cerebrolysin 10 mL vials. The price for a 100 mL saline solution vial was worked out by gathering the prices for 20 vial boxes from 3 independent sources (the websites of major distributors of pharmaceutical products in Romania) and averaging the per-vial price. The resulting price describes the theoretical daily cost of the Cerebrolysin add-on therapy from the CARS study to the standard treatment of ischemic stroke. Costs were then converted across currencies based on the referenced conversion rate and rounded to the nearest single decimal value. Costs for acute discharges below 21 days were estimated at 150 RON (30 EUR) per day for infusion. An annual discount rate of 3% was applied to acute care costs.

### 2.3. Calculating the Incremental Cost-Effectiveness Ratio of Cerebrolysin Add-on Therapy for Ischemic Stroke Using a Deterministic Approach

Having computed the costs described above, we calculated the incremental cost-effectiveness ratios (ICERs) for the Cerebrolysin add-on therapy compared to the standard treatment of ischemic stroke from the CARS study: 30 mL per day, diluted in physiological saline solution up to a total of 100 mL, for 21 days. The formula used for calculating the incremental cost-effectiveness ratios in all scenarios was:

$$\frac{\left[cost\;of\;added\;Cerebrolysin\;\left(A\right)\;-\;cost\;of\;standard\;treatment\;\left(B\right)\right]}{\left[QALYs\;Cerebrolysin\;\left(C\right)\;-\;QALYs\;placebo\;\left(D\right)\right]}$$

We calculated three *ICER* values, one for each of the three different health state valuation models used for generating health utilities based on which *QALYs* were calculated. 

### 2.4. Probabilistic Sensitivity Analysis

Probabilistic sensitivity analysis (PSA) was performed to deal with parameter uncertainty and the hypothetical nature of the scenarios and inputs used for deterministic ICER calculations. We used a custom MS Excel spreadsheet to perform 10,000 ICER simulations per patient pathway and represented these on cost-effectiveness planes. Cost-effectiveness acceptability curves (CEACs) were constructed by plotting probabilities for the cost-effectiveness of the intervention against threshold increments up to 50,000 EUR. PSA random sampling was performed based on log normal (QALYs) or uniform (costs) distributions. The highest and lowest aggregate values by health state valuation model were used as the upper and lower bounds of QALY distributions for patient groups.

## 3. Results

We compared baseline characteristics (demographics, stroke severity, social and medical history) of the 193 patients for whom we calculated QALYs. No major differences were found, allowing for a relevant comparison of health outcomes between the Cerebrolysin and placebo groups. 

The QALYs calculated using the utilities resulting from SF-36 to SF-6D conversion were aggregated by health state valuation model: standard gamble (0.698 Cerebrolysin vs. 0.643 placebo), ordinal (0.704 Cerebrolysin vs. 0.647 placebo) and Bayesian posterior mean (0.653 Cerebrolysin vs. 0.613 placebo). Average total costs per patient were 7214 RON (1458 EUR) for Cerebrolysin and 5197 RON (1050 EUR) for placebo. This amounts to a mean increment in treatment cost difference between patients treated with Cerebrolysin and standard care of 2017 RON (408 EUR), as highlighted in Table 1, and a mean increment in QALYs between 0.01–0.015, depending on the valuation technique (Table 2).

Three different ICER values corresponding to the health state valuation models used are presented in Table 2. The lowest ICER values were determined based on the ordinal valuation (18,788 EUR/QALY), while the highest were concluded for the Bayesian posterior valuation method (26,981 EUR/QALY).

### Probabilistic Sensitivity Analysis

**Figure 2:**
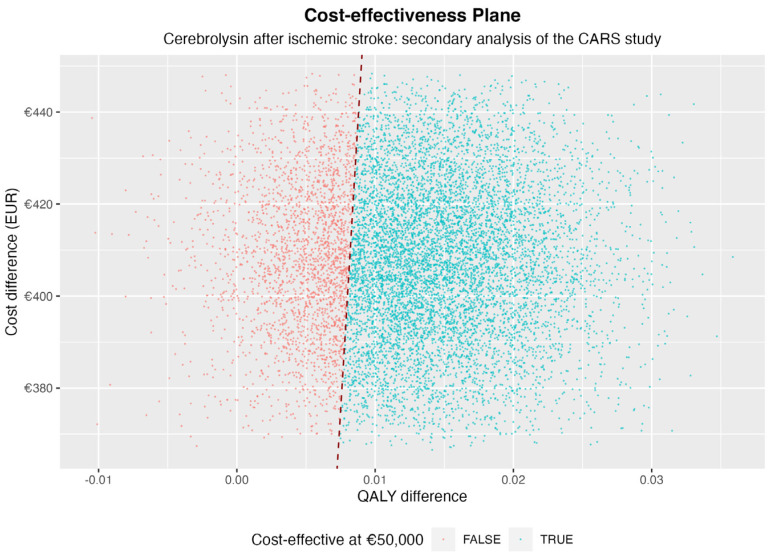
Cost-effectiveness plane, highlighting the favorable cost-effectiveness of Cerebrolysin against placebo under a 50,000 EUR threshold (red dashed line); axes represent differences in incremental cost in EUR (vertical axis) and utility (horizontal axis) between intervention and standard treatment.

**Figure 3:**
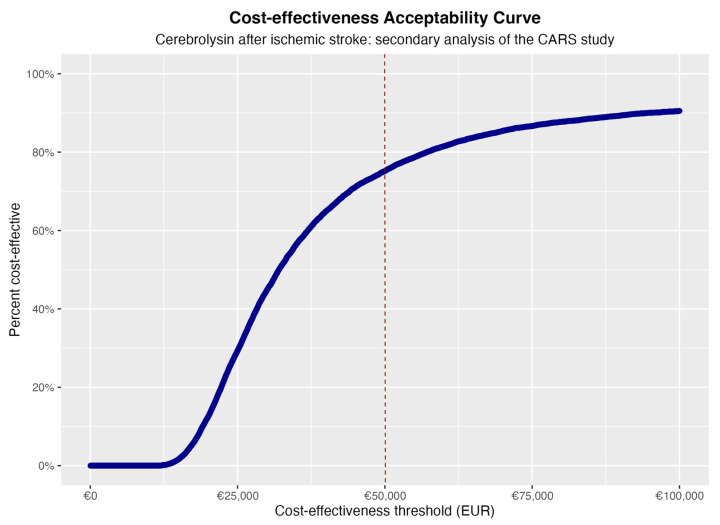
Cost-effectiveness acceptability curve (CEAC), highlighting probability of cost-effectiveness (vertical axis, percentual) at various monetary thresholds (horizontal axis, EUR).

Based on the CARS trial patient pathway, the Cerebrolysin add-on treatment was cost-effective in roughly 80% of cases for an ICER threshold set at 50,000 EUR (Figure 2 and Figure 3), considering only the 90-day timeframe of the CARS-study.

## 4. Discussion

In this study, we aimed to appraise the cost-effectiveness of Cerebrolysin add-on therapy compared to standard care for acute ischemic stroke by calculating ICER values for different cost scenarios using deterministic and probabilistic methods. Assuming that Cerebrolysin add-on therapy generates no additional costs with extra hospitalization, we concluded the agent to be cost-effective 80% of the time considering a high willingness-to-pay threshold. Our findings are in line with the scarce existing literature on this topic [32,33], providing much-needed additional evidence of the economic viability of this therapy. The next step would be to conduct a budget impact analysis that would accompany the results produced by our study [39]. 

Kulikov and Abdrashitova have conducted a cost-effectiveness analysis of Cerebrolysin in moderate and severe patients and have reported life years gained (LYG) as an efficiency criterion. Their inquiry demonstrated that Cerebrolysin (vs. standard stroke therapy) is a dominant intervention. The CER (cost-effectiveness ratio) for Cerebrolysin was 2367 EUR less compared to standard stroke therapy [32]. Walter et al. [33] employed a Markov-model on a horizon of 10 years from the payer’s perspective and used mRS data collected (from multiple sources) at day 90 post-stroke and reported health benefits in terms of QALYs and life years (LYs). This study demonstrated that Cerebrolysin is cost-effective as it reduces costs incurred in acute care and nursing homes. However, the difference between Cerebrolysin and rt-PA vs. rt-PA are slightly higher for the first scenario (3.77 vs. 3.75 QALYs).

Additionally, our examination includes the CARS treatment methodology, which involves an extended duration of hospitalization to conclude the 21-day Cerebrolysin regimen. These circumstances apply to healthcare environments where the guidance from expert organizations to establish comprehensive physical rehabilitation initiatives following an ischemic stroke [40,41] is adhered to. Within this framework, we have found that Cerebrolysin proves to be cost-effective in 80% of cases when considering a higher willingness-to-pay limit. Conversely, a lower threshold renders the intervention less financially viable, indicating that providing Cerebrolysin in an outpatient setting after prompt discharge would serve as a suitable alternative to prolonging hospital stays solely for Cerebrolysin administration, which would impose an unwarranted fiscal burden on healthcare budgets.

To our knowledge, this study yields the first explicit measure of change in QALY values between standard therapy and Cerebrolysin add-on scheme for ischemic stroke in the first year after the event. Aggregated QALYs for the Cerebrolysin group were higher than those for the placebo group (0.051 average difference across the three health state valuation models), indicating that the Cerebrolysin add-on scheme has a beneficial impact on quality of life of ischemic stroke patients. The SF-36 to utility converter from the University of Sheffield provides three options for generating health utility scores: standard gamble, ordinal and Bayesian posterior mean. Each of these approaches employs a different health state valuation model to transform SF-36 data into utility scores. The standard gamble method is based on the concept of risk preference, wherein individuals are asked to choose between a certain health outcome and a gamble with a probability of either perfect health or a less desirable outcome. This method captures the individual’s preferences and risk tolerance in relation to different health states. The ordinal approach, on the other hand, ranks health states according to their desirability, without considering the magnitude of preference between them. This approach is simpler to implement but may not capture the full spectrum of preference differences between health states. Lastly, the Bayesian posterior mean model combines prior beliefs and observed data to generate health utility scores. This method accounts for uncertainty and can adaptively update utility scores as new data become available. Using all three valuation models and comparing their results can offer valuable insights into the robustness and sensitivity of the derived utility scores. By doing so, we can assert that there are no potential variations in preferences and risk attitudes which may impact the overall cost-effectiveness analysis.

Our findings should be interpreted in conjunction with the selected study setting. The Romanian healthcare system, based on social health insurance, features significant government involvement, with the Ministry of Health (MoH) playing a central role in regulation, control and coordination. Public Health Directorates at the county level implement MoH directives and oversee local healthcare tasks. The MoH also coordinates other governmental healthcare institutions, such as the National Health Insurance House, the National Agency for Medicines and Medical Devices and the National Authority for Quality Management in Health Care. Local public authorities and the Romanian Parliament also influence the healthcare system. In Romania, the healthcare system heavily emphasizes inpatient care, with insufficient focus on primary, ambulatory and preventive care. Access to healthcare remains a challenge, particularly in rural areas, leading to high levels of unmet medical needs. Stroke care in Romania involves many stakeholders, including emergency medicine, intensive care, neurology, neurosurgery, radiology and rehabilitation. The pre-hospital phase is managed by the Ambulance Service and the Mobile Emergency Service for Resuscitation (SMURD). Patients are transported to hospitals with designated stroke acute care units. The rehabilitation process begins in the neurology ward or in a dedicated rehabilitation unit [42]. In 2015, the MoH introduced the “Priority Action-Stroke” (PAS) program to finance reperfusion treatment for acute ischemic stroke and endovascular treatment for subarachnoid hemorrhage. Initially covering 10 hospitals, the program expanded to 44 hospitals performing intravenous thrombolysis (IVT) by 2021. However, only a few hospitals can perform endovascular thrombectomy (EVT) for stroke patients, while the rest can only conduct intravenous thrombolysis. Notably, most hospitals are not meeting comprehensive stroke unit standards, as defined by the European Stroke Organization (ESO) [43]. From 2015 to 2018, around 1800 patients received reperfusion treatment through the PAS program, as reported by the Romanian Ministry of Health. The percentage of patients receiving IVT treatment was 0.8% in 2017. By 2022, 3242 patients received reperfusion treatment, accounting for 7.8% of all ischemic stroke patients. This marks a significant improvement but falls short against the estimate of patients eligible for pharmacological reperfusion and the ESO’s 15% recommended minimum rate for IVT. In 2021, only 0.9% of patients underwent EVT, well below the estimated 10% of eligible patients and the ESO’s 5% target for 2030 [44].

Cerebrolysin is reimbursed in Romania in the acute setting and in the open pharmaceutical circuit under list B (50% reimbursement). This means that, currently, there is a combination of uses and protocols within its market authorization of a 10–50 mL daily dosage for ischemic stroke. Therefore, the budget impact of normalizing care in line with the CARS protocol is impossible to measure without a high-quality observational study to assess how and how much Cerebrolysin is currently being used. An opportunity to explore this topic will emerge upon completion of the C-REGS 2 registry study [45]. Nevertheless, based on incident strokes reported by the Romanian Society of Neurology in 2022 (*n* = 37.225), a gross assumption that around 80% of cases would fit the ischemic indication [46], and of those, two-thirds of cases would be moderate–severe and hence, eligible for Cerebrolysin treatment, the incremental cost difference from our study (408 EUR) and the estimated budget impact of treating acute stroke patients for 21 days with Cerebrolysin in line with the CARS protocol would be roughly 8.5 million EUR per year, assuming the intervention would not currently exist on the market, which is around 5% of the healthcare economic burden of stroke in the country [43,47]. In addition to this proportionally small estimated budget impact (which we stress is likely overestimated in such gross calculations), given that overall stroke expenditure in Romania is up to tenfold less than other countries in Europe and the current increasing trend of expenditure at the national level [46,48], we assert that the normalization of Cerebrolysin treatment in line with the CARS protocol would entail a manageable budget impact for the Romanian healthcare system.

The limitations of our study predominantly arise from the care setting in which Cerebrolysin outcomes were determined and on which the costing exercise was based. While a 21-day treatment course is more than plausible, the guideline recommendation that Cerebrolysin is administered for a minimum of 10 days [23] allows for an assessment of the cost-effectiveness of this agent for shorter treatment courses. Such analyses would further enlighten our perspective on the economic viability of Cerebrolysin and could constitute strong evidence for its wide adoption, even in health systems with less abundant resources. The information surfaced by our study, although building a favorable cost-effectiveness profile for Cerebrolysin, calls for further economic evaluations of this treatment in different care settings along various other patient pathways and exploring issues such as the impact of the intervention on direct medical cost, recurrence and effects beyond the 90-day span of the CARS trial.

These limitations are balanced by the broadness of our methodological approach and set of tools employed. Firstly, health utilities for Cerebrolysin and standard of care have been derived using three different health state valuation models, consequently resulting in three types of ICER values. Cost data was extracted from the same care setting which utilities were derived from, hence, decreasing overall uncertainty. Furthermore, our probability sensitivity analysis accounts for several patient profiles and pathways in this specific care setting. Data from another study assessing cost of inpatient stroke care in the same exact setting [47] rendered similar values for daily hospitalization as our costing exercise did. The theoretical cost of the Cerebrolysin treatment course has been built to reflect its highest possible value. The strongest asset of our approach, though, consists of the simultaneous use of deterministic and probabilistic methods. The deterministic method produces concrete values that can be referenced against specific ICER thresholds, even though there is much debate around the way willingness-to-pay thresholds are established and used [49]. The probabilistic method encompasses diverse scenarios for costs and outcomes valid in both resource-abundant and resource-constrained health systems and provides a wide perspective on the economic implications determined by Cerebrolysin usage.

The emergence of health systems from the pandemic paradigm surfaces an imperative for more consistent use of economic evidence in healthcare decision-making and resource allocation. As the burden of stroke is estimated to increase in the following decades [50], strategies for stroke care need to be carefully crafted and regularly revised so that they strike a balance between producing health gains and remaining sustainable. Such an approach requires perpetual assessment of the cost-effectiveness of both existing and emergent therapies for stroke based on newly formed evidence on costs and outcomes.

Our results indicate a favorable cost-effectiveness profile for Cerebrolysin add-on treatment in moderate–severe acute ischemic stroke based on the CARS treatment paradigm at a 50,000 EUR cost-effectiveness threshold. Cost-effectiveness thresholds are used to determine whether an intervention is considered cost-effective in comparison to a relevant alternative, such as the standard of care. These thresholds typically represent the maximum amount a decision-maker, such as a healthcare payer, is willing to pay for an additional unit of health outcome, such as a quality-adjusted life year (QALY). It is important to note that there is no universally agreed-upon threshold, as the appropriate value depends on the specific context, including the health care system, the country’s willingness to pay and the disease area being studied. 

In our sensitivity analysis, we chose a 50,000 EUR threshold as the maximum threshold in line with the definition of high-value interventions from the Institute for Clinical and Economic Review’s Value Assessment Framework [51]. Nevertheless, we acknowledge that different stakeholders may have different thresholds based on their priorities and resources. To account for this variability, we used cost-effectiveness acceptability curves (CEACs) to explore the likelihood of Cerebrolysin being cost-effective across a range of potential threshold values. CEACs help decision-makers understand the probability of an intervention being cost-effective at different willingness-to-pay thresholds, allowing for a more flexible and context-specific interpretation of the results. This approach helps convey the uncertainty in our analysis and allows stakeholders to make informed decisions based on their unique circumstances and priorities. Our result must be interpreted against the objectives of health systems. Despite several attempts to reform Health Technology Assessment [52], Romania still uses a scorecard system based on assessments from other countries [53]. Therefore, a cost-effectiveness threshold has not been set by the public payer. While previous works from the World Health Organization (WHO-CHOICE) have suggested such thresholds may be set in line with per capita gross domestic product, there is wide criticism of such recommendations as they have no link with willingness to pay [54]. While priorities in the Romanian health system and the population’s health-related preferences are a rich ground for debate, we argue the threshold itself if not relevant in the context of this trial-based cost-effectiveness analysis, as QALY gain is underestimated over the chronic phase of stroke that follows after the initial 90 days of treatment. The question whether Cerebrolysin would be cost-effective in Romania under the CARS protocol remains difficult to answer in the absence of a decision-analytic model that accounts for a longer post-stroke timespan. Nevertheless, this analysis paves the way for future economic evaluations of this agent performed in different contexts, countries and from various other perspectives. It simultaneously adds to the evidence base to be used for shaping future strategies aimed at effectively and sustainably reducing the currently heavy societal burden of stroke.

## Figures and Tables

**Table 1:** Descriptive values of total medical cost (EUR) used in the analysis.

Total Medical Cost Estimates	Cerebrolysin	Standard Treatment
Sample size	100	93
Mean	1458 EUR	1050 EUR
Median	1466 EUR	1767 EUR
Lower threshold (95%)	1437 EUR	1029 EUR
Upper threshold (95%)	1478 EUR	1070 EUR
Standard deviation	106 EUR	101 EUR

**Table 2:** Mean QALYs derived from CARS SF-36 data for 90 days after stroke by valuation technique.

Valuation Technique	QALYs Derived from CARS		Deterministic ICER (EUR)
	Cerebrolysin	Standard Treatment	
Bayesian posterior	0.166	0.156	€26,981
Ordinal	0.182	0.168	€18,788
Standard gamble	0.186	0.171	€20,618

## Data Availability

Cost and utility data used for this analysis is available at Strilciuc, Stefan, 2023, “Replication Data for: Cost-Effectiveness of Cerebrolysin after Ischemic Stroke: Secondary Analysis of the CARS Study”, https://doi.org/10.7910/DVN/PCRGJE, Harvard Dataverse, V1.

## References

[1] Feigin V.L., Stark B.A., Johnson C.O., Roth G.A., Bisignano C., Abady G.G., Abbasifard M., Abbasi-Kangevari M., Abd-Allah F., Abedi V. (2021). Global, Regional, and National Burden of Stroke and Its Risk Factors, 1990–2019: A Systematic Analysis for the Global Burden of Disease Study 2019. Lancet Neurol..

[2] Foreman K.J., Marquez N., Dolgert A., Fukutaki K., Fullman N., McGaughey M., Pletcher M.A., Smith A.E., Tang K., Yuan C.-W. (2018). Forecasting Life Expectancy, Years of Life Lost, and All-Cause and Cause-Specific Mortality for 250 Causes of Death: Reference and Alternative Scenarios for 2016–40 for 195 Countries and Territories. Lancet.

[3] Herpich F., Rincon F. (2020). Management of Acute Ischemic Stroke. Crit. Care Med..

[4] Stinear C.M., Lang C.E., Zeiler S., Byblow W.D. (2020). Advances and Challenges in Stroke Rehabilitation. Lancet Neurol..

[5] Al-Jishi A., Saluja R.S., Al-Jehani H., Lamoureux J., Maleki M., Marcoux J. (2011). Primary or Secondary Decompressive Craniectomy: Different Indication and Outcome. Can. J. Neurol. Sci..

[6] Diener H.C., Rothwell P.M. (2022). Antithrombotic Drugs in Secondary Stroke Prevention: Still Some Way to Go. Lancet.

[7] Kalseth J., Halvorsen T. (2020). Health and Care Service Utilisation and Cost over the Life-Span: A Descriptive Analysis of Population Data. BMC Health Serv. Res..

[8] Jayawardana S., Cylus J., Mossialos E. (2019). It’s Not Ageing, Stupid: Why Population Ageing Won’t Bankrupt Health Systems. Eur. Heart J. Qual. Care Clin. Outcomes.

[9] Webb E., Hernández-Quevedo C., Williams G., Scarpetti G., Reed S., Panteli D. (2022). Providing Health Services Effectively during the First Wave of COVID-19: A Cross-Country Comparison on Planning Services, Managing Cases, and Maintaining Essential Services. Health Policy.

[10] Winkelmann J., Webb E., Williams G.A., Hernández-Quevedo C., Maier C.B., Panteli D. (2022). European Countries’ Responses in Ensuring Sufficient Physical Infrastructure and Workforce Capacity during the First COVID-19 Wave. Health Policy.

[11] Second Round of the National Pulse Survey on Continuity of Essential Health Services during the COVID-19 Pandemic. https://www.who.int/publications-detail-redirect/WHO-2019-nCoV-EHS-continuity-survey-2021.1.

[12] White T.G., Martinez G., Wang J., Gribko M., Boltyenkov A., Arora R., Katz J.M., Woo H.H., Sanelli P.C. (2021). Impact of the COVID-19 Pandemic on Acute Ischemic Stroke Presentation, Treatment, and Outcomes. Stroke Res. Treat..

[13] Meza H.T., Lambea Gil Á., Saldaña A.S., Martínez-Zabaleta M., Juez P.D.L.R., Martínez E.L.C., Apilánez M.C., Isasi M.H., Enguita J.M., Alfonso M.D.L. (2020). Impact of COVID-19 Outbreak on Ischemic Stroke Admissions and in-Hospital Mortality in North-West Spain. Int. J. Stroke.

[14] Nogueira R.G., Qureshi M.M., Abdalkader M., Martins S.O., Yamagami H., Qiu Z., Mansour O.Y., Sathya A., Czlonkowska A., Tsivgoulis G. (2021). Global Impact of COVID-19 on Stroke Care and IV Thrombolysis. Neurology.

[15] Zhao J., Li H., Kung D., Fisher M., Shen Y., Liu R. (2020). Impact of the COVID-19 Epidemic on Stroke Care and Potential Solutions. Stroke.

[16] Nawabi N.L.A., Duey A.H., Kilgallon J.L., Jessurun C., Doucette J., Mekary R.A., Aziz-Sultan M.A. (2022). Effects of the COVID-19 Pandemic on Stroke Response Times: A Systematic Review and Meta-Analysis. J. NeuroInterv. Surg..

[17] The Territorial Impact of COVID-19: Managing the Crisis and Recovery across Levels of Government. https://www.oecd.org/coronavirus/policy-responses/the-territorial-impact-of-covid-19-managing-the-crisis-and-recovery-across-levels-of-government-a2c6abaf/.

[18] McKee M., Suhrcke M., Nolte E., Lessof S., Figueras J., Duran A., Menabde N. (2009). Health Systems, Health, and Wealth: A European Perspective. Lancet.

[19] Langhorne P., Ramachandra S. (2020). Stroke Unit Trialists’ Collaboration Organised Inpatient (Stroke Unit) Care for Stroke: Network Meta-Analysis. Cochrane Database Syst. Rev..

[20] Neumann P.J., Ganiats T.G., Russell L.B., Sanders G.D., Siegel J.E., Neumann P.J., Ganiats T.G., Russell L.B., Sanders G.D., Siegel J.E. (2016). Cost-Effectiveness in Health and Medicine.

[21] Drummond M.F., Sculpher M.J., Claxton K., Stoddart G.L., Torrance G.W. (2015). Methods for the Economic Evaluation of Health Care Programmes.

[22] Bettger J.P., Cadilhac D.A. (2022). Stroke Care Costs and Cost-Effectiveness to Inform Health Policy. Stroke.

[23] Beghi E., Binder H., Birle C., Bornstein N., Diserens K., Groppa S., Homberg V., Lisnic V., Pugliatti M., Randall G. (2021). European Academy of Neurology and European Federation of Neurorehabilitation Societies Guideline on Pharmacological Support in Early Motor Rehabilitation after Acute Ischaemic Stroke. Eur. J. Neurology.

[24] Platz T. (2020). Rehabilitative Therapy for Arm Paresis Following a Stroke. https://www.awmf.org/leitlinien/detail/ll/080-001.html.

[25] Teasell R., Hussein N., Mirkowski M., Vanderlaan D., Saikaley M., Longval M., Iruthayarajah J. (2020). Stroke Rehabilitation Clinician Handbook.

[26] Muresanu D.F. (2009). Neuroplasticity and Neurorecovery. Stroke.

[27] Strilciuc S. (2021). Safety of Cerebrolysin for Neurorecovery after Acute Ischemic Stroke: A Systematic Review and Meta-Analysis of Twelve Randomized-Controlled Trials. Pharmaceuticals.

[28] Bornstein N.M., Guekht A., Vester J., Heiss W.-D., Gusev E., Hömberg V., Rahlfs V.W., Bajenaru O., Popescu B.O., Muresanu D. (2018). Safety and Efficacy of Cerebrolysin in Early Post-Stroke Recovery: A Meta-Analysis of Nine Randomized Clinical Trials. Neurol. Sci..

[29] Alvarez X.A., Sampedro C., Figueroa J., Tellado I., González A., García-Fantini M., Cacabelos R., Muresanu D., Moessler H. (2008). Reductions in QEEG Slowing over 1 Year and after Treatment with Cerebrolysin in Patients with Moderate-Severe Traumatic Brain Injury. J. Neural. Transm..

[30] Muresanu D.F., Florian S., Hömberg V., Matula C., von Steinbüchel N., Vos P.E., von Wild K., Birle C., Muresanu I., Slavoaca D. (2020). Efficacy and Safety of Cerebrolysin in Neurorecovery after Moderate-Severe Traumatic Brain Injury: Results from the CAPTAIN II Trial. Neurol. Sci..

[31] Poon W., Matula C., Vos P.E., Muresanu D.F., von Steinbüchel N., von Wild K., Hömberg V., Wang E., Lee T.M.C., Strilciuc S. (2019). Safety and Efficacy of Cerebrolysin in Acute Brain Injury and Neurorecovery: CAPTAIN I-a Randomized, Placebo-Controlled, Double-Blind, Asian-Pacific Trial. Neurol. Sci..

[32] Kulikov A., Abdrashitova G. (2015). Cost-Effectiveness Analysis of Cerebrolysin In The Treatment of Patients With Acute Ischemic Stroke Moderate and Severe Degrees of Severity In The Russian Federation. Value Health.

[33] Walter E., Bauer M., Ressl S. (2015). Cost-Effectiveness Of Combined Treatment With Alteplase (Rt-Pa) And Cerebrolysin In Acute Ischemic Hemispheric Stroke In Austria. Value Health.

[34] Vladescu C., Scintee S.G., Olsavszky V., Hernandez-Quevedo C., Sagan A. (2016). Romania: Health System Review. Health Syst. Transit..

[35] GDP per Capita (Current US$)—Romania, Data. https://data.worldbank.org/indicator/NY.GDP.PCAP.CD?locations=RO.

[36] Muresanu D.F., Heiss W.-D., Hoemberg V., Bajenaru O., Popescu C.D., Vester J.C., Rahlfs V.W., Doppler E., Meier D., Moessler H. (2016). Cerebrolysin and Recovery After Stroke (CARS): A Randomized, Placebo-Controlled, Double-Blind, Multicenter Trial. Stroke.

[37] Calculating QALYs from the SF-36 and SF-12, Available from University of Sheffield. https://licensing.sheffield.ac.uk/product/SF-6D.

[38] Bernhardt J., Hayward K.S., Kwakkel G., Ward N.S., Wolf S.L., Borschmann K., Krakauer J.W., Boyd L.A., Carmichael S.T., Corbett D. (2017). Agreed Definitions and a Shared Vision for New Standards in Stroke Recovery Research: The Stroke Recovery and Rehabilitation Roundtable Taskforce. Int. J. Stroke.

[39] Mauskopf J.A., Sullivan S.D., Annemans L., Caro J., Mullins C.D., Nuijten M., Orlewska E., Watkins J., Trueman P. (2007). Principles of Good Practice for Budget Impact Analysis: Report of the ISPOR Task Force on Good Research Practices—Budget Impact Analysis. Value Health.

[40] Winstein C.J., Stein J., Arena R., Bates B., Cherney L.R., Cramer S.C., Deruyter F., Eng J.J., Fisher B., Harvey R.L. (2016). Guidelines for Adult Stroke Rehabilitation and Recovery. Stroke.

[41] Ringelstein E.B., Chamorro A., Kaste M., Langhorne P., Leys D., Lyrer P., Thijs V., Thomassen L., Toni D. (2013). European Stroke Organisation Recommendations to Establish a Stroke Unit and Stroke Center. Stroke.

[42] (2018). Acțiunea Prioritară Pentru Tratamentul Intervențional Al Pacienților Cu Avc Acut Procedură Operațională Standard Privind Traseul Pacientului Și Protocolul Terapeutic.

[43] Strilciuc S., Grad D.A., Mixich V., Stan A., Buzoianu A.D., Vladescu C., Vintan M.A. (2021). Societal Cost of Ischemic Stroke in Romania: Results from a Retrospective County-Level Study. Brain Sci..

[44] Norrving B., Barrick J., Davalos A., Dichgans M., Cordonnier C., Guekht A., Kutluk K., Mikulik R., Wardlaw J., Richard E. (2018). Action Plan for Stroke in Europe 2018–2030. Eur. Stroke J..

[45] Vester J., Bornstein N., Heiss W.-D., Vosko M., Moessler H., Jech M., Winter S., Brainin M. (2021). C-REGS 2—Design and Methodology of a High-Quality Comparative Effectiveness Observational Trial. J. Med. Life.

[46] Tiu C., Terecoasă E.O., Tuță S., Bălașa R., Simu M., Sabău M., Stan A., Radu R.A., Tiu V., Cășaru B. (2023). Quality of Acute Stroke Care in Romania: Achievements and Gaps between 2017 and 2022. Eur. Stroke J..

[47] Lorenzovici L., Székely A., Csanádi M., Gaál P. (2020). Cost Assessment of Inpatient Care Episodes of Stroke in Romania. Front. Public Health.

[48] Strilciuc S., Grad D.A., Radu C., Chira D., Stan A., Ungureanu M., Gheorghe A., Muresanu F.-D. (2021). The Economic Burden of Stroke: A Systematic Review of Cost of Illness Studies. J. Med. Life.

[49] Thokala P., Ochalek J., Leech A.A., Tong T. (2018). Cost-Effectiveness Thresholds: The Past, the Present and the Future. Pharmacoeconomics.

[50] Wafa H.A., Wolfe C.D.A., Emmett E., Roth G.A., Johnson C.O., Wang Y. (2020). Burden of Stroke in Europe. Stroke.

[51] Value Assessment Framework. https://icer.org/our-approach/methods-process/value-assessment-framework/.

[52] Lopert R., Ruiz F., Gheorghe A., Chanturidze T. (2017). Technical Assistance for institution building of Health Technology Assessment structure, including training for the National Agency for Medicines & Medical Devices. Situational Analysis of Romanian HTA.

[53] Radu C.-P., Chiriac N.D., Pravat A.M. (2016). The Development of the Romanian Scorecard HTA System. Value Health Reg. Issues.

[54] McDougall J.A., Furnback W.E., Wang B.C.M., Mahlich J. (2020). Understanding the Global Measurement of Willingness to Pay in Health. J. Mark. Access Health Policy.

